# Springs vs. motors: Ideal assistance in the lower limbs during walking at different speeds

**DOI:** 10.1371/journal.pcbi.1011837

**Published:** 2024-09-04

**Authors:** Israel Luis, Maarten Afschrift, Elena M. Gutierrez-Farewik

**Affiliations:** 1 KTH MoveAbility, Department of Engineering Mechanics, KTH Royal Institute of Technology, Stockholm, Sweden; 2 Faculty of Behavioural and Movement Sciences, VU Amsterdam, Amsterdam, The Netherlands; 3 Department of Women’s and Children’s Health, Karolinska Institutet, Stockholm, Sweden; Universidade do Lisboa Faculdade de Motricidade Humana, PORTUGAL

## Abstract

Recent years have witnessed breakthroughs in assistive exoskeletons; both passive and active devices have reduced metabolic costs near preferred walking speed by assisting muscle actions. Metabolic reductions at multiple speeds should thus also be attainable. Musculoskeletal simulation can potentially predict the interaction between assistive moments, muscle-tendon mechanics, and walking energetics. In this study, we simulated devices’ optimal assistive moments based on minimal muscle activations during walking with prescribed kinematics and dynamics. We used a generic musculoskeletal model with tuned muscle-tendon parameters and computed metabolic rates from muscle actions. We then simulated walking across multiple speeds and with two ideal actuation modes–motor-based and spring-based–to assist ankle plantarflexion, knee extension, hip flexion, and hip abduction and compared computed metabolic rates. We found that both actuation modes considerably reduced physiological joint moments but did not always reduce metabolic rates. Compared to unassisted conditions, motor-based ankle plantarflexion and hip flexion assistance reduced metabolic rates, and this effect was more pronounced as walking speed increased. Spring-based hip flexion and abduction assistance increased metabolic rates at some walking speeds despite a moderate decrease in some muscle activations. Both modes of knee extension assistance reduced metabolic rates to a small extent, even though the actuation contributed with practically the entire net knee extension moment during stance. Motor-based hip abduction assistance reduced metabolic rates more than spring-based assistance, though this reduction was relatively small. Our study also suggests that an assistive strategy based on minimal muscle activations might result in a suboptimal reduction of metabolic rates. Future work should experimentally validate the effects of assistive moments and refine modeling assumptions accordingly. Our computational workflow is freely available online.

## Author summary

We used simulation to identify ideal assistance at major lower limb joints that can potentially be produced by motor-based or spring-based assistive devices in slow, normal, and fast walking. We found that assistance from both actuation modes decreased muscle activations and net muscle moments to varying extents, depending on joint and walking speed, but they did not always reduce metabolic energy of muscles. Motor-based assistance was overall more effective than spring-based assistance, and spring-based assistance at times increased the metabolic energy. The largest metabolic energy reductions occurred with motor-based plantarflexion assistance, followed by motor-based hip flexion assistance, both more notably at higher speeds. Motor-based hip abduction assistance also reduced metabolic energy, somewhat inversely with walking speed. Spring-based assistance was overall less effective than motor-based assistance but did reduce metabolic energy with plantarflexion assistance in slow walking and with hip flexion assistance in fast walking. Knee extension assistance, regardless of actuation mode or walking speed, had little to no influence on metabolic energy. Our simulation findings do not support knee extension assistance at all, nor spring-based hip flexion assistance in slow walking or hip abduction assistance at any speed if a device goal is to reduce muscle activations.

## 1. Introduction

Multiple lower limb exoskeletons have made breakthroughs in the past decade by improving walking and running efficiency [1]. Increasingly efficient actuators and batteries, better strategies for human-device control, and lighter structures and physical interfaces have continuously improved assistance efficiency [2]. Current efforts to bridge the gap between laboratory-based observations and real-world benefits frequently focus on refining methods to identify optimal assistance [3], integrating human movement intention into exoskeleton control [4], and expanding exoskeleton use to make them suitable across multiple locomotion modes [5]. In this regard, musculoskeletal simulations can complement these efforts by guiding hypotheses about muscle-device interaction and revealing causal relationships in experimental observations [6].

Prior musculoskeletal simulation studies of exoskeleton assistance have provided insights into muscle-tendon mechanics and energetics, though simulation findings have not always agreed with experimental observations. Researchers have, through simulations, estimated the influence of exoskeleton assistance on tendon energy storage and release [7], on muscle fiber operating lengths and velocities [8], and on muscle activations, all of which influence muscle energetics and metabolic rates [9–11]. In theory, a model-based approach can be used to design exoskeleton controllers that result in optimal muscle dynamics and minimal energy cost. For instance, Franks et al. [12] used simulations with prescribed kinematics and dynamics to predict optimal multi-joint assistive moments, i.e. leading to minimal metabolic rates during walking. In subsequent experiments with these assistive moments, they indeed observed reduced muscle excitations and metabolic costs, but not as much as the model predicted. Uchida et al. [10] used a similar computational approach to predict optimal assistive moments for running; these were later evaluated experimentally by Lee et al. [13], who reported decreased metabolic cost, but again not as much as the model predicted. Some discrepancies between simulations and experiments are to be expected, as modeling approaches rely on a number of assumptions, including simplified muscle control and dynamics, simplified or no user-device interaction forces, massless devices, and unchanged kinematics. Model-based approaches thus have potential use in informing the design of assistive interventions, more so if they can accurately estimate muscle energetics and metabolic rates.

Most musculoskeletal modeling studies aiming to predict optimal assistive moments have focused on gait at or near preferred walking speed, even though daily activities encompass a wide range of speeds and locomotion modes. Several studies have predicted optimal lower limb exoskeleton assistive moments near preferred walking speed in normal and loaded conditions, such as carrying extra weight [6,11]. Other activities, such as walking at various speeds or stair ascent, have been studied less [6]. To the best of our knowledge, only two musculoskeletal modeling-based studies have examined optimal assistance during gait at a range of speeds; Uchida et al. examined mechanics and energetics in young adults with ideal actuators during running at 2 and 5 m/s [10], and Cseke et al., in elderly adults during walking at self-selected slow (0.86 m/s), comfortable (1.22 m/s) and fast (1.53 m/s) speeds [9].

Whereas variable assistive torque profiles that can theoretically be provided by a motor can be expected to reduce metabolic rates, spring-based actuation, i.e., a spring or elastic component that can store potential energy during elongation and then release it during shortening, can also potentially influence muscle dynamics and gait energetics. Spring-based exoskeletons can also potentially be lighter and less cumbersome than motorized exoskeletons. Spring-based assistance should theoretically be effective during gait phases characterized by joint power absorption followed by joint power generation, which is the case with ankle dorsi-/plantarflexion and hip flex-/extension in preswing, hip ab-/adduction during midstance, and knee flex-/extension during loading response and midstance [14]. Spring-based actuators have been observed experimentally to substantially decrease physiological joint moments, i.e., joint moments from muscle actions, but with negligible metabolic reduction [15]. Prior musculoskeletal simulation studies have provided insights into the causal relationship between muscle mechanics and spring-based assistive moments near preferred speed [16,17] predictions have agreed with experimental observations to some degree [18,19]. Simulations that investigate the influence of spring-based assistance on muscle energetics can potentially inform device design.

The objectives of the study were thus to simulate how two modes of assistance, spring-based and motor-based, at individual lower limb joints, affect computed muscle dynamics and metabolic rates during walking at various speeds. We hypothesized that assistive moments will reduce muscle activations and metabolic rates and that motor-based actuation will be more effective than spring-based actuation—the ability to generate a versatile profile with net positive power from the motor-based actuation will outperform the kinematic-constraint zero-net power assistance from spring-based actuation. Also, assisting ankle plantarflexion with any mode of assistance will yield the largest reduction of metabolic rates compared to unassisted conditions among all the muscle groups and at all walking speeds.

## 2. Methods

### 2.1 Musculoskeletal simulation workflow

**Fig 1:**
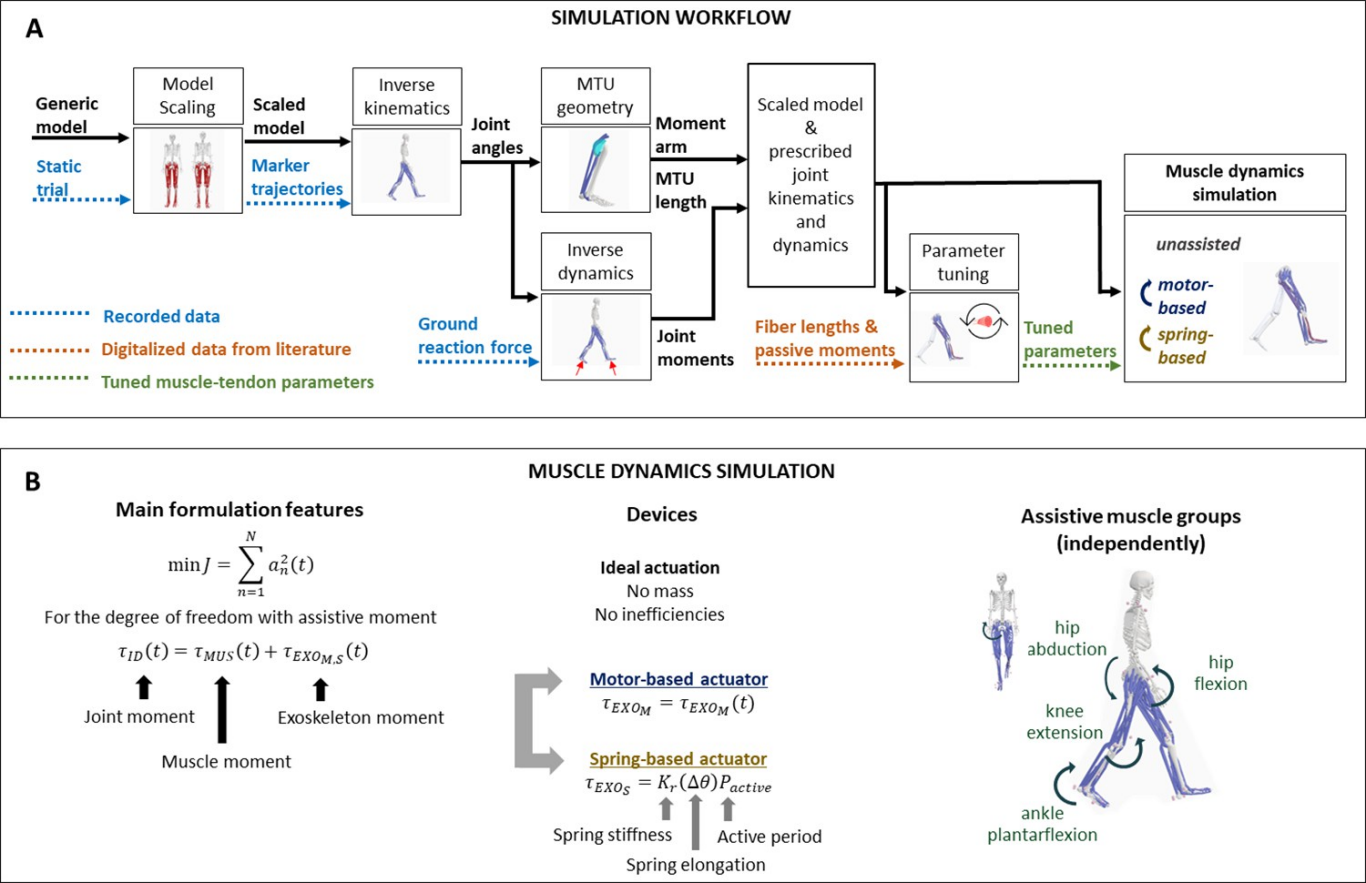
Simulation scheme. Simulation workflow (Fig 1A) and muscle dynamic simulation (Fig 1B) for each subject. Inverse kinematics and dynamics are computed using the OpenSim workflow. Moment arms and muscle-tendon lengths are computed from the inverse kinematic solution using the Muscle Analysis tool from OpenSim. We then tuned the muscle-tendon parameters–optimal fiber length, tendon slack length, and tendon stiffness–such that the simulated muscle fiber lengths and excursions matched reported findings from ultrasound imaging reported in the literature. Next, we tuned the muscle passive force-length relationships such that the simulated passive moments matched joint passive moment-angle relationships from an *in vivo* study reported in the literature. Finally, we simulated walking across various speeds with no actuators and with the various assistive actuators.

We implemented a simulation workflow to estimate muscle dynamics and metabolic rates during walking using musculoskeletal models with tuned muscle-tendon parameters and recorded motion. We used previously reported experimental data [20] and the OpenSim software [21] to scale a generic musculoskeletal model and compute joint kinematics and dynamics. We tuned muscle-tendon parameters in the scaled musculoskeletal model to better represent fiber lengths and joint passive moment-angle relationships [22]. Then, we performed musculoskeletal simulations while walking with prescribed kinematics and dynamics using trajectory optimization [23] (Fig 1). Detailed descriptions of the experimental data, computational methods, and data analysis are provided in the following subsection.

### Experimental data

Experimental motion data: marker trajectories and ground reaction force of five unimpaired (2/3 male/female, [mean ± SD] age: 31.4 ± 7.4 years old, height: 1.75 ± 0.03 m, body mass: 69.0 ± 10.3 kg), collected at the Promobilia MoveAbility Lab, reported in a previous publication were used for this study [20]. In brief, subjects walked on a treadmill at a range of walking speeds, specifically 55%, 70%, 85%, 100%, 115%, 130%, and 145% of their estimated preferred walking speed (PWS). Subjects then walked along a lab pathway and emulated different walking speeds by matching recorded cadences from treadmill walking. Oxygen and carbon dioxide rates were recorded during treadmill trials, and motion data were recorded during overground trials. Regarding the overground trials, marker positions (100 Hz), based on the Conventional Gait Model with the Extended-foot model (CGM 2.4) described by Leboeuf et al. [24], and ground reaction forces (1000 Hz) were measured using optical motion capture (Vicon V16, Oxford, UK) and strain gauge force platforms (AMTI, Watertown, MA, USA), respectively.

### Musculoskeletal model, joint kinematics, and inverse dynamics

A generic musculoskeletal model developed by Rajagopal et al. [25] with modified hip abductor muscle paths [26] was selected for this study. We scaled the generic model using OpenSim’s Scale Tool, which adjusted muscle-tendon unit paths, skeletal geometries, and segment inertial properties to fit anthropometric dimensions obtained from a captured static calibration trial. We adjusted the maximum isometric force of the soleus, gastrocnemius lateralis, gastrocnemius medialis, and tibialis anterior, as per Arnold et al. [27], to 3586 N, 606 N, 1308 N, and 674 N, respectively. These are lower than the original isometric forces in the model from Rajagopal et al., which were 6195 N, 1575 N, 3116 N, and 1227 N, respectively, and resulted in estimated muscle excitation magnitudes that better resemble recorded EMGs normalized by maximum isometric contraction, reported in the literature [28]. Estimated muscle excitation magnitudes of other muscles, such as vastus lateralis or gluteus medius, were similar to normalized EMG and, thus, not adjusted [28].

Marker trajectories and ground reaction forces throughout three gait cycles per subject at low (55% PWS), normal (100% PWS), and fast (145% PWS) walking speeds were analyzed with inverse kinematics and inverse dynamics using OpenSim 4.1. Marker tracking weights for inverse kinematics were selected to minimize the error between experimental and virtual markers. The subtalar and metatarsal joints were fixed at neutral anatomical positions.

### Tuning of muscle-tendon parameters

We used a computational tool to tune muscle-tendon parameters such that each subject’s muscle excitations, fiber lengths, and passive moments best resembled experimental observations [22]. The tuning was done in two steps. First, we tuned optimal fiber lengths, tendon slack lengths, and tendon stiffnesses of the gastrocnemius lateralis, gastrocnemius medialis, soleus, and vasti (lateralis, medialis, and intermedius) to match muscle fiber lengths and excursions obtained from those reported in from ultrasound imaging [29,30]. Then, we tuned muscle passive force-length relationships to match the reported passive moment at various ankle, knee, and hip joint angles from an *in vivo* study [31]. Compared to simulation with the original generic model, these steps result in estimated muscle excitations that better agree with observed electromyography signals reported in the literature [22].

### Solving muscle redundancy

Our implementation is based on the simulation framework proposed by De Groote et al. [23], which uses direct collocation dynamic optimization and implicitly incorporates activation and contraction dynamics. Specifically, contraction dynamics, formulated using normalized muscle fiber length as a state and introducing a scaled time derivative of the normalized muscle length as a new control, was imposed as a constraint in the optimization problem. Muscle excitations, states, and state derivatives were computed based on the assumption that the muscle redundancy is solved by the optimization criterion of minimum sum of squared muscle activations. Two other terms are present in the objective function to improve the feasibility and convergence of the formulation. Reserve actuators in each degree of freedom are added to guarantee the problem’s feasibility, and their use is penalized in the objective function. Also, a term that minimizes muscle fiber velocities is included to improve numerical computation. The objective function is implemented as (1):

$$min(w_{a}\int_{t_{i}}^{t_{f}}\sum_{n=1}^{N}a_{n}^{2}(t)dt++w_{r}\int_{t_{i}}^{t_{f}}\sum_{j=1}^{J}e_{R_{j}}^{2}(t)dt+w_{v}\int_{t_{i}}^{t_{f}}\sum_{n=1}^{N}{\tilde{v}}_{n}^{2}(t)dt)$$ (1)

Where *a*_*n*_ is muscle activation of muscle *n*, $e_{R_{j}}$ is the excitation of the reserve actuator of joint *j*, ${\tilde{v}}_{n}$ is the normalized fiber velocity of muscle *n*, *t*_*f*_ and *t*_*i*_ are the initial and final times of the gait cycle, respectively; *N* and *J* are the total number of muscles and joints in the musculoskeletal model, respectively; and *w*_*a*_, *w*_*r*_ and *w*_*v*_ are the weights of the terms in the objective function related to the muscle activations, reserve actuators, and fiber velocities, respectively. The sum of the moments produced by muscle-tendon and reserve actuators equals the net joint moment obtained from inverse dynamics at each joint. This condition was implemented as a constraint in the optimization problem as in (2)

$$\tau_{ID_{j}}(t)=\tau_{{MUS}_{j}}(t)+e_{R_{j}}(t)T_{R},j=1,\ldots ,J$$ (2)

Where, at joint *j*, $\tau_{ID_{j}}$ is the net joint moment, $\tau_{{MUS}_{j}}$ is the moment produced by the muscle-tendon actuators, subsequently referred to here as “muscle moments”, and *T*_*R*_ is the magnitude of the reserve actuator.

### 3.2 Determining optimal assistive moments

Optimal assistive moments were computed using scaled musculoskeletal models with tuned muscle-tendon parameters, inverse kinematics, and inverse dynamics solutions as described above. Constraints and design variables were added when solving the muscle redundancy to model assistive moments at various muscle groups. For each mode of actuation, ideal assistive moments were added at each simulated gait cycle to assist specific muscle groups individually: plantarflexion, knee extension, hip flexion, and hip abduction. Per each subject, nine gait cycles were simulated (three gait cycles per walking speed); therefore, nine assistive moments per mode of actuation and muscle group were determined.

When solving the muscle redundancy, the objective function was the same as in unassisted conditions, but constraints were added to model assistive device moment to assist muscle-tendon actuators in reproducing the inverse dynamics, i.e., the sum of the muscle moment, reserve actuator moment, and assistive device moment equals the net joint moment at each joint, as in (3)

$$\tau_{ID_{j}}(t)=\tau_{{MUS}_{j}}(t)+e_{R_{j}}(t)T_{R}+\tau_{{EXO_{M,S}}_{j}}(t),j=1,\ldots ,J$$ (3)

Where $\tau_{{EXO_{M,S}}_{j}}$ is the assistive device moment at joint *j*. In this regard, the assistive moments are the optimal solutions to assist muscles based on minimal summed of squared muscle activations.

#### Motor-based moment profiles

The motor-based actuation was modeled as a unidirectional ideal moment at the corresponding degree of freedom in the musculoskeletal model. The assistive moment ($\tau_{EXO_{M}}(t)$) was implemented as a time-series design variable. Its magnitude was constrained to assist the aimed muscle group explicitly. For instance, $\tau_{EXO_{M}}(t)<0$ for assisting ankle plantarflexion corresponds to ankle plantarflexion moment (agonist muscle group) and avoids generating ankle dorsiflexion moment (antagonist muscle group). The motor-based actuation was not constrained in its trajectory; hence, it could have any value at each point in time to assist a muscle group. Optimal assistive moment based on motor-based actuation was determined individually for each subject and gait cycle.

#### Spring-based device parameters

The spring-based actuator was modeled as a unidirectional torsional spring that engages and disengages in specific joint angles. To implement this, we introduced three design variables: engaged timing (*t*_*c*_), disengaged timing (*t*_*d*_), and spring stiffness (*k*_*r*_), we added a constraint to impose that the angle at which the spring engaged and disengaged are similar as in (4)

$${\left(q(t_{c})-q(t_{d})\right)}^{2}<0.01$$ (4)

Where *q*(*t*) is the joint angle corresponding to the assisted muscle group. The assistive moment was computed as a product of the spring stiffness and the angle within the period that the spring is engaged as in (5)

$$\tau_{EXO_{S}}(t)=k_{r}q_{EXO}(t)$$ (5)

Where *q*_*EXO*_(*t*) is the joint angle displacement from the angle of engagement. This angle was modeled using hyperbolic tangent function as in (6), (7), (8), and (9)

$$P_{e}(t)=0.5+0.5\;\tanh\left(b(time(t)-t_{c})\right)$$ (6)

$$P_{d}(t)=0.5+0.5\;\tanh\left(b(t_{d}-time(t))\right)$$ (7)

$$P_{active}(t)=P_{e}(t)P_{d}(t)$$ (8)

$$q_{EXO}(t)=P_{active}(t)(q(t)-q(t_{c}))$$ (9)

Where *P*_*e*_ is the start of the engaged period, *P*_*d*_ is the start of the disengagement period, and *P*_*active*_(*t*) is the period where spring is engaged.

We selected *w*_*a*_, *w*_*r*_ and *w*_*v*_ as 1, 1000, and 0.001; thus, the use of reserve actuators was heavily penalized, and the influence of fiber velocities was relatively small. Also, we selected *T*_*R*_ as 100 Nm, and b as 1000 since it provided a smooth yet steep transition between null to assistive moment generation (S1 Fig). Optimal assistive moment based on spring-based actuation was determined individually for each subject and gait cycle.

### 3.3 Metabolic rate computation

For each subject/gait cycle, each speed, and each device, the metabolic rate of each muscle was computed based on the muscle excitations, states, and state derivatives obtained from our optimization routine using a metabolic energy model proposed by Bhargava et al. [32], which we previously reported to agree with recorded metabolic rates obtained from spiroergonometry [20]. In brief, muscle metabolic rate is computed as in (10)

$${\dot{E}}_{n}(t)={\dot{W}}_{CE_{n}}(t)+{\dot{H}}_{n}(t)$$ (10)

Where ${\dot{E}}_{n},\;{\dot{W}}_{CE_{n}}$ and ${\dot{H}}_{n}$ are the metabolic rate, contractile element work rate, and heat rate, respectively, of muscle *n*. The contractile element work rate, also called muscle power, is computed as in (11)

$${\dot{W}}_{CE_{n}}(t)=F_{CE_{n}}(t)V_{CE_{n}}(t)$$ (11)

Where $F_{CE_{n}}$ and $V_{CE_{n}}$ are the muscle force and fiber velocity, respectively, of muscle *n*. The heat rate depends on muscle mass, muscle activations, fiber velocities, and a function that approximates the size principle of motor recruitment, explained in detail by Bhargava et al. [32]. The original formulation did not explicitly address negative metabolic rates, which are possible during eccentric contractions if muscle negative power exceeds the heat rate. As a negative metabolic rate is physiologically questionable, we adjusted it in such cases by updating the heat rate and re-computing the metabolic rate as in (12) and (13)

$${\dot{H}}_{n,mod}(t)=-{\dot{W}}_{CE_{n}}(t)-{\dot{H}}_{n}(t),{\dot{E}}_{n}(t)<0$$ (12)

$${\dot{E}}_{n}(t)={\dot{W}}_{CE_{n}}(t)+{\dot{H}}_{n,mod}(t)$$ (13)

The metabolic rates for one leg (${\dot{E}}_{L}$) is computed as the sum of all the individual muscle metabolic rates as in (14)

$${\dot{E}}_{L}(t)=\sum_{n=1}^{N}{\dot{E}}_{n}(t)$$ (14)

### F. Data and statistical analysis

We evaluated the change of physiological joint moments, muscle activations, and metabolic rates between unassisted and assisted with two actuation modes during walking across speeds. We computed the average physiological agonist and antagonist net joint moments at each joint with the device’s assistance, i.e., ankle, knee, and hip joints in the sagittal plane and hip joint in the frontal plane. Net muscle moments are defined here as the net joint moments minus the assistive moments (3). We computed the average times-series of the muscle activations and metabolic rates over all the muscles in one leg and over all the muscles that span the assisted (plantarflexion, knee extension, hip flexion, and hip abduction) and antagonist (dorsiflexion, knee flexion, hip extension, and hip adduction) muscle group as in (15)

$$X(t)=\frac{1}{S}\sum_{s=1}^{S}x_{s}(t),s\in [1,2,\ldots ,S]$$ (15)

Where *X* is the average time-series of muscle activations and metabolic rates in one leg, and the assisted and antagonist muscle groups composed of all the muscles *x*_*s*_. The value of the index *s* considers either all muscles, i.e., represents average values accounting for all muscles in one leg, or muscles that span the assisted or antagonist muscle group. For each speed, we presented the average time-series values of the net moment, muscle activation, and metabolic rates in the assisted and antagonist muscle group in unassisted and assisted conditions, e.g., with ideal plantarflexion assistive moments, average times-series values of the plantarflexion and dorsiflexion muscle activations, net muscle moments, and metabolic rates in unassisted conditions and with motor-based and spring-based actuation at slow, normal, and fast walking speed were presented.

Also, we computed the average values over each gait cycle of the muscle activations, net muscle moment, and metabolic rates in one leg and the assisted and antagonist muscle groups. This metric was calculated as the integral of its corresponding time-series (previously calculated in (15)) divided by the gait cycle duration as in (16)

$$\bar{X}=\frac{1}{t_{f}-t_{i}}\int_{t_{i}}^{t_{f}}X\;dt$$ (16)

Where $\bar{X}$ is the average value over a gait cycle of the average muscle activations, net muscle moment, and metabolic rates in one leg, and the assisted and antagonist muscle groups. To facilitate comparison, we computed the change (Δ) in the average values over a gait cycle of the muscle activations, net muscle moment, and metabolic rates for each gait cycle between unassisted (${\bar{X}}_{unassisted}$) and assisted (${\bar{X}}_{assisted}$) conditions and presented it as a percentage of that value in unassisted conditions as in (17)

$$\Delta =\frac{{\bar{X}}_{unassisted}-{\bar{X}}_{assisted}}{{\bar{X}}_{unassisted}}x\;100\%$$ (17)

For each walking speed, we presented the change in average metabolic rates vs. change in average muscle activations in one leg between unassisted and assisted muscle groups and the change in net muscle moments, muscle activations, and metabolic rates in the assisted and antagonistic muscle groups between unassisted and assisted muscle groups.

In addition, to complement the description of the estimated muscle-tendon mechanics and energetics, we presented the activations, work rates (obtained from (11)), and metabolic rates of individual muscles for unassisted and assisted conditions at normal walking speed.

## 3. Results

### 3.1 Influence of assistive moments on relative muscle activations and metabolic rates

**Fig 2:**
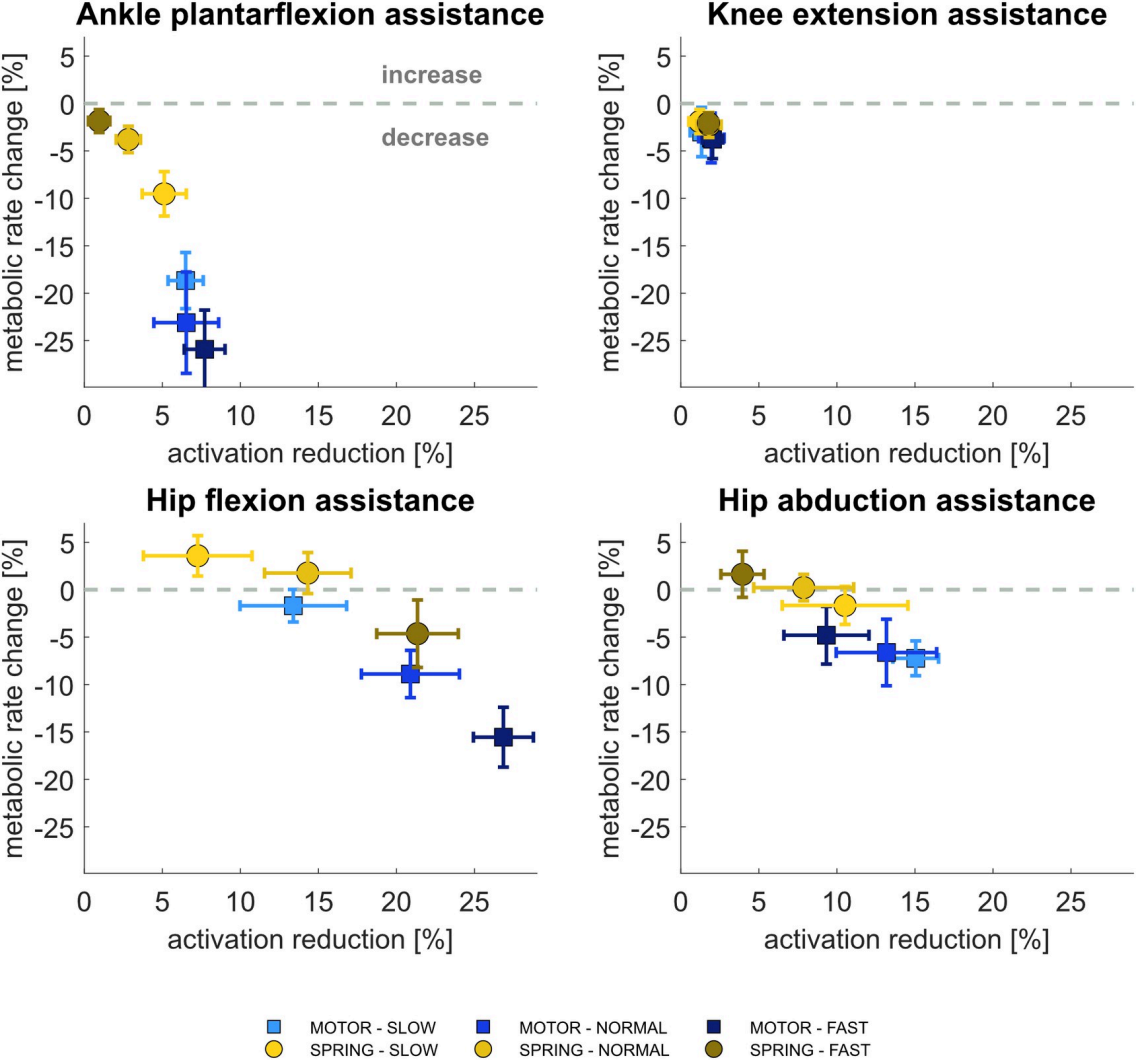
Change in metabolic rates vs. reduction of muscle activations, shown as % of unassisted conditions, at slow, normal, and fast walking speeds with motor-based and spring-based assistance. The values shown are average ± 1 standard deviation among all subjects and gait cycles.

Compared to unassisted conditions, with either actuation mode, relative muscle activation changes varied, depending on the joint and muscle group assisted and with walking speed (Figs 2 and S2). With motor-based actuation, muscle activation reduced most overall with hip flexion assistance at a high walking speed; this change decreased with decreasing walking speeds. The next highest muscle activation reduction was observed with hip abduction assistance, which, in contrast to hip flexion assistance, was proportionally higher as walking speed decreased. Muscle activations were reduced moderately with plantarflexion assistance, with a small relation to walking speed. Muscle activations were nearly unchanged with knee extension assistance at any walking speed.

With spring-based actuation, relative muscle activations were nearly with identical trends as with motor-based actuation, though all proportionally lower, with one major contrast, that muscle activation changes with plantarflexion assistance were inversely proportional to walking speed, and were practically zero at fast walking speed.

While relative muscle activation changes were largely proportional to relative metabolic rate changes, they did not always translate to reduced metabolic cost; spring-based assistance actually resulted in 2–4% higher metabolic rates, most notably with hip flexion assistance at slow and normal speeds and with hip abduction assistance at fast speed. The largest reduction (average ca. 7%) of relative metabolic rate with spring-based actuation resulted from ankle plantarflexion assistance at slow speed, followed ca. 5% reduction with hip flexion reduction at fast speed.

Motor-based assistance always caused a decrease in metabolic rates (S2 Fig), wherein the highest relative reduction (average ca. 24%) was observed with ankle plantarflexion assistance at fast speed, followed by ankle plantarflexion assistance at lower speeds (22% at normal and 16% at slow speeds) then by hip flexion assistance (15%) at high walking speed. Hip abduction assistance reduced metabolic rates somewhat (average ca. 7%) at normal speed, varying slightly with speed. Hip flexion assistance at low speed had practically no effect on metabolic rate change, nor did knee extension assistance at any speed.

Analyses of the influence of ideal assistive moments at each joint are described in more detail in the next section.

### 3.2. Ankle plantarflexion assistance

**Fig 3:**
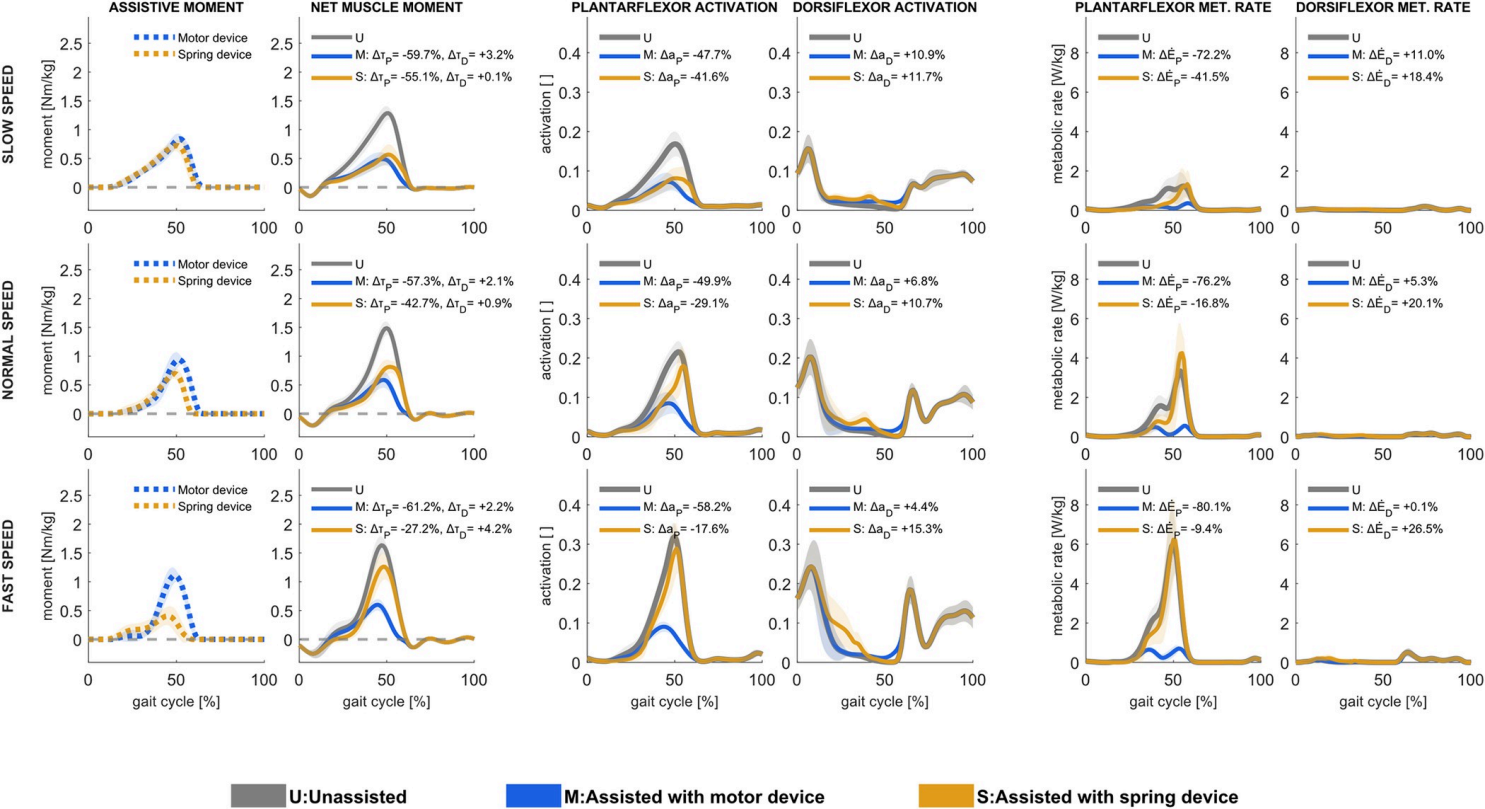
Assistive device moments [first column], net muscle moments [second column], muscle activations in ankle plantarflexors [third column] and ankle dorsiflexors [fourth column], and metabolic rates in ankle plantarflexors [fifth column] and ankle dorsiflexors [sixth column] in unassisted conditions and with motor-based and spring-based assistance during slow (upper row), normal (middle row), and fast (lower row) walking speed. Mean values ± 1 standard deviation among all subjects and gait cycles are illustrated. Positive moment refers to ankle plantarflexion, and negative to ankle dorsiflexion. Change in ankle plantarflexion (Δτ_P_) and ankle dorsiflexion (Δτ_D_) moments, ankle plantarflexor (Δa_P_) and ankle dorsiflexor (Δa_D_) muscle activations, and ankle plantarflexor ($\Delta {\dot{E}}_{P}$) and ankle dorsiflexor ($\Delta {\dot{E}}_{D}$) muscles’ metabolic rates, shown as % of unassisted conditions, are presented.

The computed ideal motor-based plantarflexion assistance contributed with more than half of the net ankle plantarflexion moment and only increased slightly in magnitude with increasing speed; the net plantarflexion muscle moment was reduced by approximately 60% at all speeds (Fig 3), while the net dorsiflexor muscle moment increased by up to 4%. With motor-based assistance, the total metabolic rate peak at all speeds was reduced near terminal stance and preswing phases. Overall, these differences resulted in a 16% reduction in overall metabolic rate in slow walking and a 24% reduction in fast walking. Soleus activation was nearly entirely reduced with motor-based plantarflexion assistance, and, to a lower extent, gastrocnemius activation (S3 Fig). The gastrocnemius still generated a moment during midstance, contributing to the ankle plantarflexion and knee flexion moments. The activation of dorsiflexion muscles during midstance, specifically the anterior tibialis, was nearly the same in unassisted conditions.

Ideal spring-based plantarflexion assistance contributed with more overall moments in slow walking than in normal or fast walking; the plantarflexor muscle moment was reduced by more than half (55%) in slow walking, by 43% in normal and 27% in fast walking. The peak ankle dorsiflexion angle, which sets the assistive moment peak, occurs earlier in the gait cycle as walking speed increases; the spring can thus not maximally assist the muscle plantarflexor moment peak at preswing to the same extent as motor-based actuation can. During terminal stance, plantarflexion muscle activations, specifically soleus and gastrocnemius, were reduced with spring-based assistance, but dorsiflexion muscle activations were increased. Muscle fiber velocities increased in the soleus and gastrocnemius during push-off, and, as a result, muscle positive power increased (S3 and S4 Figs), resulting in increased plantarflexion metabolic rate peak at all speeds even though the average metabolic rate over the gait cycle decreased (S5 Fig).

### 3.3. Knee extensor assistance

**Fig 4:**
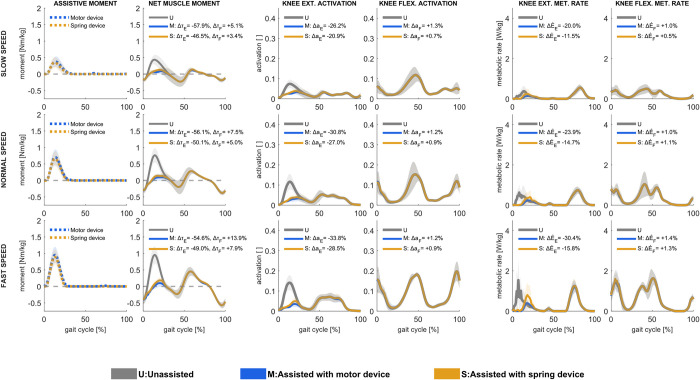
Assistive device moments [first column], net muscle moments [second column], muscle activations in knee extensors [third column] and knee flexors [fourth column], and metabolic rates in knee extensors [fifth column] and knee flexors [sixth column] in unassisted conditions and with motor-based and spring-based assistance during slow (upper row), normal (middle row), and fast (lower row) walking speed. Mean values ± 1 standard deviation among all subjects and gait cycles are illustrated. Positive moment refers to knee extension, and negative to knee flexion. Change in knee extension (Δτ_E_) and knee flexion (Δτ_F_) moments, knee extensor (Δa_*E*_) and knee flexor (Δa_*F*_) muscle activations, and knee extensor ($\Delta {\dot{E}}_{E}$) and knee flexor ($\Delta {\dot{E}}_{F}$) muscles’ metabolic rates, shown as % of unassisted conditions, are presented.

Ideal motor-based knee extensor assistance was only effectual in loading response and early midstance, where it contributed with nearly all knee extensor moments at all walking speeds (Fig 4). The assistive moment resulted in a net muscle moment decrease of 47–50% at all speeds. The assistive moment resulted in a slightly increased knee flexion moment just after initial contact, more so at high walking speed. With assistance, during loading response, vasti activations decreased, but muscle power increased (S3 and S4 Figs); knee extension assistance resulted in decreased vasti tendon force, which decreased tendon strain and thus increased fiber excursions and velocities. As a result, both muscle negative power during loading response and muscle positive power in early midstance increased. Consequently, metabolic rates from vasti dynamics decreased in loading response and increased slightly in early midstance (S5 Fig). Overall, the motor-based assistance resulted in a 2–3% metabolic rate reduction at all walking speeds.

Ideal spring-based knee extensor assistance was likewise only effectual in loading response and early midstance, to practically the same degree as motor-based assistance. It resulted in similar reductions in muscle activations, net muscle moments, and metabolic energy rates, yet to a somewhat lower magnitude; with assistance, the total metabolic rate was reduced by approximately 2% at all speeds.

### 3.4. Hip flexor assistance

**Fig 5:**
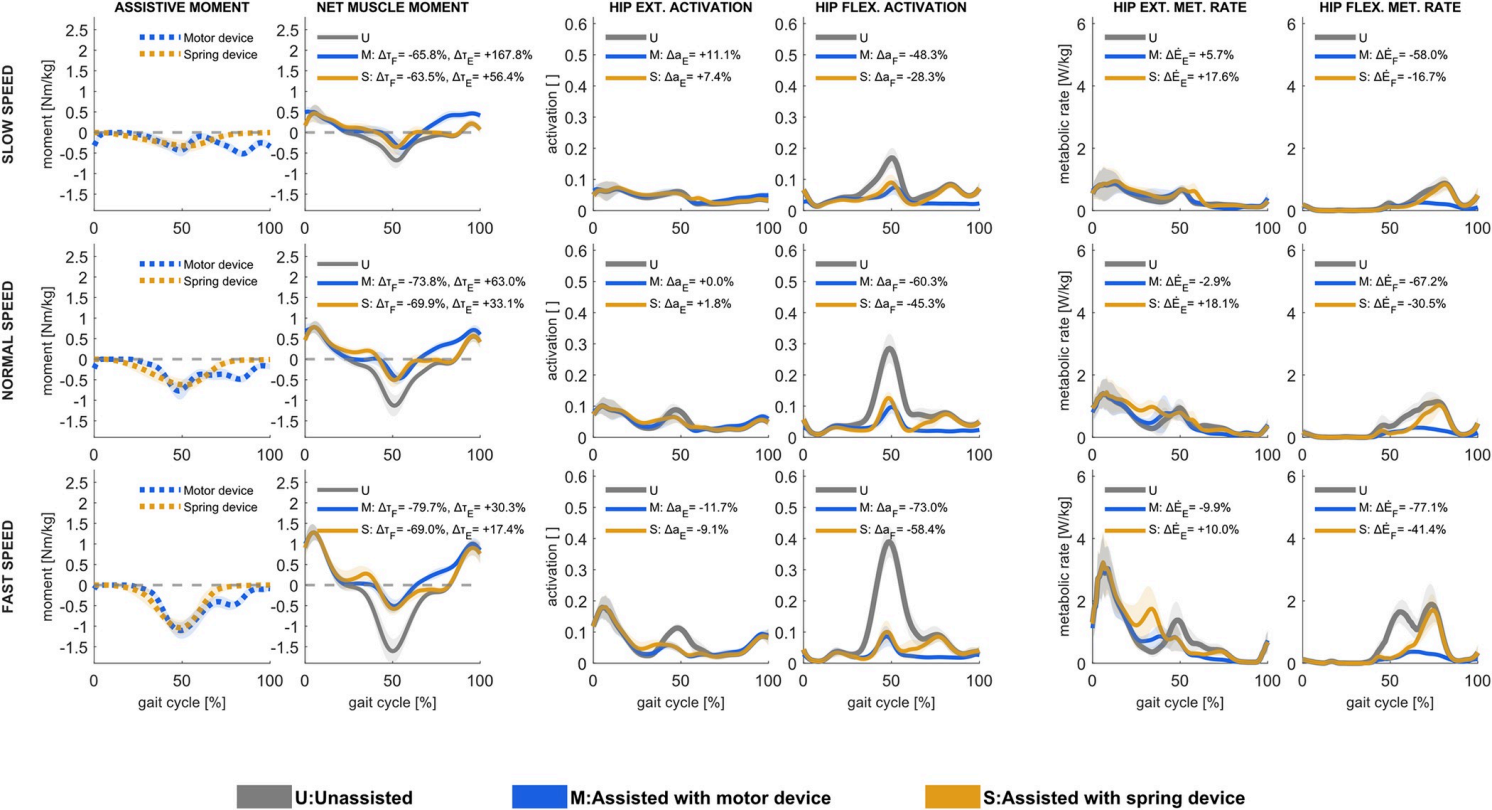
Assistive device moments [first column], net muscle moments [second column], muscle activations in hip extensors [third column] and hip flexors [fourth column], and metabolic rates in hip extensors [fifth column] and hip flexors [sixth column] in unassisted conditions and with motor-based and spring-based assistance during slow (upper row), normal (middle row), and fast (lower row) walking speed. Mean values ± 1 standard deviation among all subjects and gait cycles are illustrated. Positive moment refers to hip extension, and negative to hip flexion. Change in hip extension (Δτ_E_) and hip flexion (Δτ_F_) moments, hip extensor (Δa_*E*_) and hip flexor (Δa_*F*_) muscle activations, and hip extensor ($\Delta {\dot{E}}_{E}$) and hip flexor ($\Delta {\dot{E}}_{F}$) muscles’ metabolic rates, shown as % of unassisted conditions, are presented.

Ideal motor-based hip flexor assistance was effectual largely in terminal stance and preswing, increasing with walking speed, and mid- to late swing (Fig 5) and to a very small amount immediately after initial contact. The assistive moment resulted in substantially decreased hip flexion muscle moment, ranging from 66% reduction at slow and 80% at fast walking speeds, mostly observed in terminal stance and preswing, but also *increased* hip extensor muscle moment in mid- to late swing. The increase in hip extensor muscle moment was relatively similar at all speeds but led to a particularly remarkable 168% increase in net hip extensor muscle moment in slow walking, during which the extensor moment was negligible without assistance. The increase in hip extension muscle moment reflects a trade-off between decreased activations in the hip flexion muscle group (Figs 5 and see psoas in S3) at the expense of slightly increased activations in other muscle groups (Fig 5 and see biceps femoris long head and vastus lateralis in S3 Fig). As a result, with assistance, metabolic rates were reduced during terminal stance and preswing but increased during early to mid-swing. Without assistance, the vasti were most active during loading response and midstance, but with motor-based assistance, the vasti were also active during mid-swing, likely as antagonists for the increased biceps femoris long head activation. This activation pattern resulted in increased vasti force and power during the swing phase (S4 Fig), which caused vasti negative power during initial swing and positive power during mid-swing. As muscle positive power is associated with higher metabolic rates, motor-based assistance resulted in slightly increased metabolic rates during mid-swing.

Ideal spring-based hip flexor assistance was only effectual during terminal stance and preswing, as it is set by spring engagement as the hip extends during midstance and disengagement as the hip flexes in early swing (Fig 5). With assistance, the hip flexor muscle moment was greatly reduced during this phase; the net hip flexor muscle moment was reduced by 64 in slow and 69–70% in faster walking. However, its engagement during midstance, which accommodated energy storage during hip extension, resulted in *increased* hip extensor muscle during midstance. With assistance, activation of hip extension muscles such as gluteus maximum and semimembranosus increased in midstance, and vasti activation increased in initial swing (Figs 5 and S3), resulting in higher muscle positive power and, thereby, metabolic rates during the mid- to terminal stance. In contrast, the increased vasti activation corresponded to higher muscle negative power, which did not increase metabolic rates (S4 and S5 Figs).

### 3.5. Hip abduction assistance

**Fig 6:**
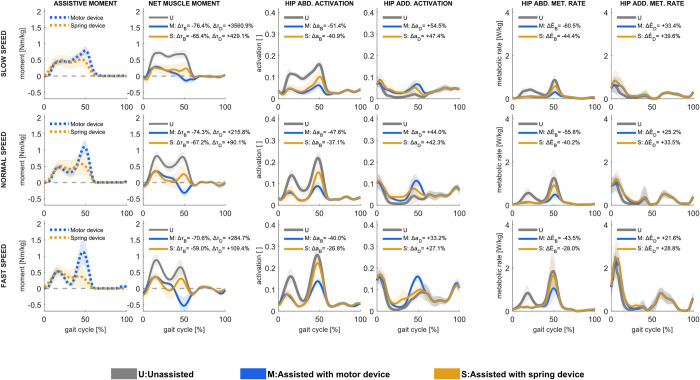
Assistive device moments [first column], net muscle moments [second column], muscle activations in hip abductors [third column] and hip adductors [fourth column], and metabolic rates in hip abductors [fifth column] and hip adductors [sixth column] in unassisted conditions and with motor-based and spring-based assistance during slow (upper row), normal (middle row), and fast (lower row) walking speed. Mean values ± 1 standard deviation among all subjects and gait cycles are illustrated. Positive moment refers to hip abduction, and negative to hip adduction. Change in hip abduction (Δτ_B_) and hip adduction (Δτ_D_) moments, hip abductor (Δa_B_) and hip adductor (Δa_D_) muscle activations, and hip abductor ($\Delta {\dot{E}}_{B}$) and hip adductor ($\Delta {\dot{E}}_{D}$) muscles’ metabolic rates, shown as % of unassisted conditions, are presented.

Ideal motor-based hip abduction assistance was effectual throughout nearly the entire stance phase, accounting for the majority of net hip abduction moment, reducing the hip abductor muscle moment by more than 70% at all walking speeds and more at slower speeds (Fig 6). The assistive moment peaked at approximately 20 and 50% of the gait cycle. Whereas the first assistive peak reduced the net muscle hip abduction moments and hip abductor muscle activations, the second peak increased the net hip adduction moment and adductor muscle activations (Figs 6 and S3), with correspondingly higher hip adductor muscle positive power and metabolic rates (S4 and S5 Figs).

Ideal spring-based hip abduction assistance was likewise effectual during nearly the entire stance phase. With spring-based assistance, the hip abductor muscle moment decreased by approximately 60% at all walking speeds. However, the overall metabolic rate was nearly unchanged; with assistance, the metabolic rate decreased by 2% in slow walking, was unchanged in normal walking, and increased by 2% in fast walking. The spring-based assistance had a less pronounced peak in terminal stance than motor-based assistance, as it was set by the hip adduction angle, and a hip abductor muscle moment was still required in this phase, though lower than without assistance. Similar to motor-based assistance, spring-based assistance involved a trade-off between decreased hip abductor muscle activation and increased hip adductor muscle activation (Figs 6 and S3). This trade-off was, however, even less effective in reducing activations and metabolic rates than the motor-based assistance. While metabolic rates decreased in gluteus medius and minimus, and tensor fasciae latae with spring-based assistance, they did not decrease as much as with motor-based assistance (S5 Fig). Also, metabolic rates in the gluteus maximum during midstance were higher with spring-based than with motor-based assistance.

## 4. Discussion

In this simulation study, ideal assistive moments were identified, defined as those that reduced the sum of squared muscle activations. The assistive moment profiles in a motor-based actuator could have a variable profile, but those with the spring-based actuators were constrained by joint kinematics. The ideal assistive moments in both actuator modes substantially decreased net muscle moments, i.e., the net joint moment minus the assistive moment. Whereas motor-based assistance always reduced total metabolic rates to some extent, varying among joints and speeds, spring-based assistance did not always reduce metabolic rates. The most notable reductions in metabolic rates resulted from motor-based plantarflexion assistance, followed by motor-based hip flexion assistance, both more effective at higher speeds. Motor-based hip abduction assistance also reduced metabolic rate, interestingly inversely with walking speed. Spring-based hip flexion assistance at slow and normal speeds and hip abduction assistance at normal and fast speeds reduced muscle activations to some extent, but these reductions did not translate to reduced metabolic rates; rates were unchanged or even increased slightly. Knee extension assistance, regardless of actuation mode or walking speed, had little to no effect on metabolic rates, even though it was able to contribute to a majority of the net extensor moment in loading response.

Our findings indicate that an assistive strategy based on minimal muscle activations does not translate to a decreased metabolic rate. Assistive devices are generally designed to support motion, which might involve reducing net muscle moment, activations, and metabolic rates [2]. The optimal assistive moments in our simulations decreased the overall sum of muscle activation, which in turn reduced muscle forces and, thus, net muscle moment in the assisted muscle groups, though occasionally increasing demand on antagonist muscles. Reduction of metabolic rates was, however, more difficult to achieve. Metabolic energy models estimate muscle energy rates based on heat dissipation and muscle power. Heat dissipation is the sum of various subcomponents that depend on fiber velocities, such as the shortening and lengthening heat rates and the activation and maintenance heat rates [32]. All components are related to muscle activations. As such, metabolic rate is diminished if heat dissipation and muscle power are zero, which only happens when muscle activations are zero. However, assistive moments that submaximally reduce muscle activations, i.e., lower but non-zero activations, can result in higher metabolic rates if the muscle positive power increase outweighs the heat dissipation decrease. We observed two instances in which this was the case, both with spring-based assistance: With plantarflexion assistance during preswing, and with knee extension assistance during midstance, wherein lower activation was associated with higher fiber velocity in the assisted muscles, which increased muscle positive power, and thereby metabolic rates. In several cases, assistive moment resulted in increased demand, and thus muscle positive power and metabolic rates, in antagonist muscles, for instance, with hip flexion assistance during mid-swing and with hip abduction assistance during loading response. It is not a straightforward assumption that assistive moments that reduce overall muscle activations will also reduce metabolic rates. We did, however, identify several cases in which the assistive moment reduced agonist muscle activations to zero, without substantially increasing antagonist muscle activations, and resulted in overall reduced metabolic rates in instances of the gait cycle, specifically with motor-based ankle plantarflexion and knee extension assistance.

Our identified ideal motor-based plantarflexion assistive moment profiles are similar to previously reported moment profiles that were found to reduce metabolic rates. At normal walking speed, the ideal motor-based plantarflexion assistive moment profile was similar to those identified from human-the-loop optimization studies that aimed for minimal metabolic rates [3,33]. The peak in the profile we identified was near 50% of the gait cycle, agreeing with other identified optimal moment trajectories [3,33]. However, our simulation predicts a metabolic reduction (22%) at normal walking speed, which is substantially larger than experimentally reported metabolic rate reduction reported by Zhang et al. at a slightly lower speed (14% metabolic reduction at 1.25 m/s) [33]. We also found that with motor-based plantarflexion assistance, the metabolic reduction should be more pronounced as walking speed increases, in agreement with prior studies [3,33]. The relative metabolic cost of plantarflexion motion respected to the total metabolic cost of the gait cycle increases with walking speeds [20]. Since motor-based plantarflexion assistance did not increase metabolic rates in antagonist or other muscle groups, it is consistent that higher metabolic saving was predicted as walking speed increased.

Our identified ideal spring-based plantarflexion assistive moments disagreed somewhat with prior reported observations. Experimental comparisons with spring-based assistance are more challenging, as very few studies have studied muscle activation changes across walking speeds. In a study from Nuckols and Sawicki using an exoskeleton emulator to mimic spring-like actuation, the optimal spring stiffness to reduce metabolic rates was similar at speeds of 1.25 and 1.75 m/s [34]. This finding does not align with our results, presumably because our optimal assistive moment does not directly minimize metabolic rates. Also, our modeled spring-based device can engage at any instance of the stance phase, while Nuckols and Sawicki engaged the spring at initial foot-ground contact.

Our findings of decreased muscle activation but increased muscle positive power during early midstance with knee extension assistance might explain why previous experimental studies with this aim have failed to reduce metabolic rates during walking. Metabolic rate reduction with knee extension assistance has only been achieved with motor-based actuation compared to wearing a powered-off exoskeleton [35,36] or in challenging environments such as carrying loads while walking on an inclined surface [37]. Vastus lateralis, a major knee extensor muscle, contracts nearly isometrically during loading response and midstance in unassisted conditions [30], which results in low metabolic rate demands. Our simulation suggests that, with knee extensor assistance, some increase in metabolic rates is observed due to the detuning of the force generation capacity associated with the fiber contraction velocity. The knee extension assistive moment decreased the knee extension muscle forces during loading response, resulting in shorter tendon elongation because the elongation of the tendons, modeled as non-linear springs [23], depends on the force they are subject to, e.g., elongation is shorter with a lower force. Furthermore, since the muscle-tendon unit length is unchanged between unassisted and assisted conditions, i.e., motion is prescribed in both conditions, shorter tendon elongation directly implies larger muscle fiber excursion. A low reduction in metabolic rates was predicted because the metabolic rate associated with knee extension is relatively small (i.e., the metabolic peak of knee extension muscles ~0.4 w/kg in unassisted conditions), and muscle fiber velocities increased during early midstance, increasing muscle positive power and metabolic rates compared to unassisted conditions. Jackson et al. reported a similar finding that even if muscle moment is reduced with an ankle exoskeleton, the metabolic cost can increase if the muscle moment corresponds to increasing muscle positive work [7]. In our study, we found little to no potential benefit from knee extensor assistance, regardless of actuation mode or walking speed.

Spring-based and especially motor-based hip flexion assistance shows promise in reducing metabolic rates, particularly at fast walking speeds. Only a few studies have evaluated the effects of hip flexion assistance with powered devices [38–40]. Studies of devices that reduced metabolic cost parametrized the assistive profile such that it began at maximum hip extension [40] or provided a power burst during a predefined time window (corresponding to 25% of the gait cycle) [39]; both studies found that the optimal peak assistive moment should be later than the net hip flexion moment. These assistive trajectories disagree somewhat with the optimal assistive moments that we identified. We also found that the increased antagonist hip extensor muscle moment during the swing phase counterintuitively reduced metabolic rate, in agreement with findings reported in another simulation study [11]. It is possible that this counterintuitive benefit from simulation studies might not translate experimentally; it has, to the best of our knowledge, not been tested experimentally. Furthermore, previous experimental studies reported a metabolic decrease of 8.8% with hip flexion assistance compared to unassisted conditions [40] and 6.1% compared with a powered-off exoskeleton [39], which agrees with our predictions (9% near preferred walking speed). With spring-based hip flexion assistance, only two experimental studies reported metabolic rate reduction [19,41]. Zhou et al. reported 7.2% metabolic rate reduction at 1.5 m/s and suggested that optimal assistive is likely speed-dependent [19]. We found no metabolic reduction with spring-based assistance at preferred walking speed but a small decrease (4%) in fast walking. To the best of our knowledge, no previous study has evaluated the influence of hip flexion assistance, neither spring- or motor-based, at different walking speeds. Our simulation supports a hypothesis that hip flexion assistance from either actuation mode can potentially decrease metabolic rate as walking speed increases.

Our findings indicate little potential for hip abduction assistance to reduce metabolic rates substantially. Recent studies have evaluated metabolic rate reduction assisting hip abduction using soft exosuits [42] and rigid frame-based exoskeletons [43]. Park et al. used a soft exosuit to explore the interaction between metabolic rates and balance based on assistive moments that mimic peak abduction moments and power [42]. They found that mimicking the physiological second peak moment (~45% of the gait cycle) reduced metabolic rates by 11% compared to unassisted conditions. Interestingly, we estimated two assistive moment peaks with motor-based assistance, both aligned with the physiological moment peaks. Yet, the first assistive moment peak increased the time-series metabolic rates of the hip adduction (antagonist) muscle group, while the second assistive moment peak (~50% of the gait cycle) decreased the time-series metabolic rates of the hip adduction muscle group. On the other hand, Kim et al. used a rigid frame-based exoskeleton to explore the metabolic reduction based on assistive moments obtained from human-in-the-loop optimization, mimicking the net hip moment in the frontal plane, and optimal assistance from musculoskeletal simulation [43]. They found none of them reduced metabolic rates compared to the unpowered exoskeleton. The simulation-based optimal assistance used by Kim et al. was based on the predicted value from Dembia et al. [11] and resembled our predicted assistive moment trajectory. In this regard, our finding suggests that predicting assistive moments based on minimal muscle activations may not be the most advantageous strategy for metabolic rate reduction. Also, our estimation suggests that applying an abduction assistive moment during ~50% of the gait cycle might decrease metabolic rates as it does not involve a substantial increase in the metabolic rate of the hip adductor muscle group across walking speeds. Future studies experimental studies might confirm or refine our observations.

The two major limitations of our study are 1) the assumption that motion patterns in unassisted and assisted conditions are unchanged, which is a dilemma in all musculoskeletal simulations with constrained kinematics, and 2) the optimal assistive moments defined with the objective function in the muscle redundancy solver that seeks the task-specific exoskeleton moments that minimize the sum of squared muscle activations. The assumption of unchanged kinematics might be reasonable in spring-based devices at the ankle [18] and hip [19], as they can be made lightweight and reasonably comfortable. With powered ankle and hip assistive devices, despite evidence suggesting that joint angles and net joint moments might be preserved [38,44], human-device adaptation is complex and more likely to alter the user’s motor control strategy and, thereby, joint kinematics and moments [45]. The motion adaptation accompanying optimal assistance can be inferred by adopting a more complex scheme, such as predictive simulation. However, enabling the prediction of joint mechanics does not necessarily imply more realism in the simulated outcome. For instance, Nikoo and Uchida showed that the predicted optimal assistance varies substantially according to the selected objective function, and the corresponding kinematic adaptation does not necessarily reflect experimentally reported values; simulation of gait with an ankle plantarflexion device and multiple objective functions [46] predicted knee flexion throughout the gait cycle which was not observed experimentally [47]. Accurate prediction of motion adaptation using exoskeleton devices might derive from a more physiologically plausible neuromuscular control model, which constitutes an active line of research. Moreover, accounting for motion adaptation is likely more relevant in conditions where assistive devices might drastically change motion patterns, such as when using devices that span multiple joints [36] or in people with motion disorders [48,49].

Regarding the second limitation, we formulated the optimization problem to solve muscle redundancy and to identify optimal assistive moments using the same objective function, specifically minimal muscle activation. We assumed the paradigm that human walking is achieved with a minimum summed of squared muscle activations, and that metabolic efficiency is driven by this neuromuscular strategy. As such, the assistive moments in our simulation represent the optimal for minimal muscle activations, but we demonstrated that reduction of muscle activations does not directly translate to reduction of metabolic rates. Our findings warrant further simulation studies that identify assistive moments with optimization goals other than minimal activations, ideally with goals of minimal metabolic cost. In this regard, it might be beneficial to explicitly incorporate the goal of the assistive moment separately from the objective function to solve muscle redundancy. This can theoretically be achieved by adopting a bilevel optimization scheme as proposed by Nguyen et al. [50], in which a low-level optimization problem might deal with solving muscle redundancy, while a upper-level problem searches for optimal assistance with a task criterion, e.g., maximal walking stability or minimal metabolic cost. Future studies might unveil unexplored human-device interaction by explicitly formulating the goals of the assistive devices.

Finally, other venues to improve our understanding of the human-device performance based on musculoskeletal simulations lie in further examining the model sensibility to predict optimal assistance. Our study aims to provide insights into the interaction between muscle dynamics and assistive moments among types of devices when assisting gait. Yet, estimations of the optimal assistance are likely subject to simplifications of the modeled musculoskeletal system [51] and human-device interface [52]. For instance, we neglected anatomical variations of the muscle-tendon geometries such as muscle-tendon length and moment arm around the hip joint, though they influence muscle forces [53] and, thus, potentially muscle energetics in unassisted and assisted conditions. Similarly, we modeled assistive devices as massless and frictionless actuators. As such, our estimation does not consider any possible inefficiency in force transmission exerted by the device to the body segments. Future studies might benefit from evaluating the sensibility of the predicted optimal assistance in correspondence with the modeled musculoskeletal geometry and human-device interface. Establishing the relationship between model selection and muscle dynamics on assistive moment might refine the prediction of the optimal assistance and guide the design of the assistive device control and structure.

## Supporting information

S1 Fig Ideal spring parameters, illustrated as angle-moment (normalized by body mass), for ankle plantarflexion [upper left], knee extension [upper right], hip flexion [lower left], and hip abduction [lower right] assistance, for each speed (columns) and subject/gait cycle (separate lines). Positive moment refers to ankle plantarflexion, knee extension, hip flexion, and hip abduction.
(TIFF)

S2 Fig Change in metabolic rates vs. reduction of muscle activations per each subject, shown as % of unassisted conditions, at slow (S), normal (N), and fast (F) walking speeds with motor-based and spring-based assistance. Each color represents a different subject (average value of three gait cycles per subject).
(TIFF)

S3 Fig Average muscle activations in unassisted conditions (gray), with motor-based (blue) and spring-based (yellow) assistance of plantarflexion [upper left corner], knee extension [upper right corner], hip flexion [lower left corner], and hip abduction [lower right corner] at normal walking speed. Muscle names (plot titles) refer to their abbreviations in the musculoskeletal model. The figure illustrates average muscle values among all the subjects and gait cycles.
(TIFF)

S4 Fig Average muscle work rate in normal conditions (gray) and with motor-based (blue) and spring-based (yellow) ankle plantarflexion [upper left corner], knee extension [upper right corner], hip flexion [lower left corner], and hip abduction [lower right corner] assistance at normal walking speed. Muscle names (plot titles) refer to their abbreviations in the musculoskeletal model. The figure illustrates average muscle values among all the subjects and gait cycles.
(TIFF)

S5 Fig Average muscle metabolic rates (normalized to body mass) in normal conditions (gray) and with motor-based (blue) and spring-based (yellow) ankle plantarflexion [upper left corner], knee extension [upper right corner], hip flexion [lower left corner], and hip abduction [lower right corner] assistance at normal walking speed. Muscle names (plot titles) refer to their abbreviations in the musculoskeletal model. The figure illustrates average values among all the subjects and gait cycles.
(TIFF)
