# Supplementary figures and images for: Springs vs. motors: Ideal assistance in the lower limbs during walking at different speeds
Source: PLoS Comput Biol. 2024 Sep 4;20(9):e1011837. doi: 10.1371/journal.pcbi.1011837 (PMC11404844; doi:10.1371/journal.pcbi.1011837)

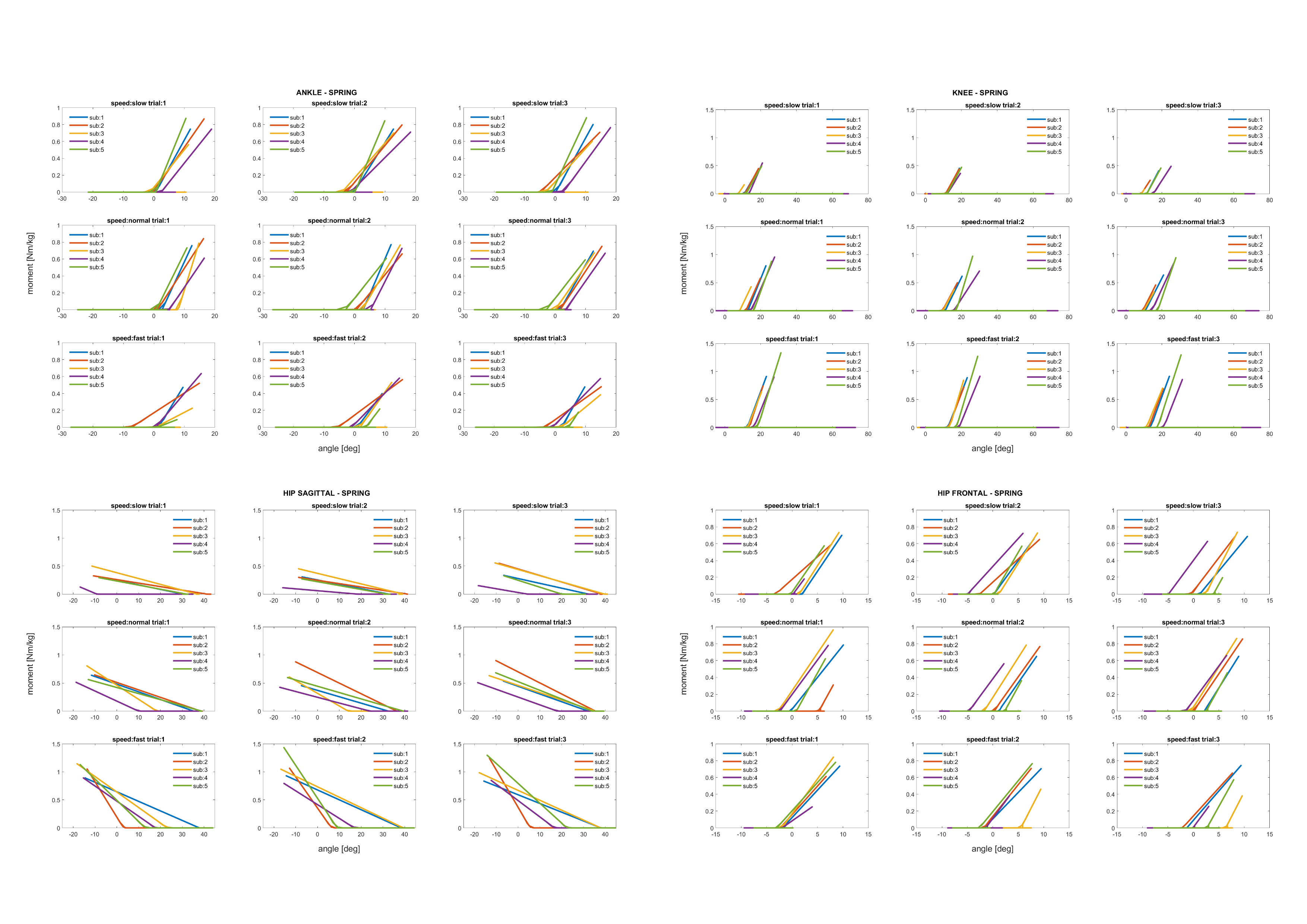

Supplement: S1 Fig — Ideal spring parameters, illustrated as angle-moment (normalized by body mass), for ankle plantarflexion [upper left], knee extension [upper right], hip flexion [lower left], and hip abduction [lower right] assistance, for each speed (columns) and subject/gait cycle (separate lines). Positive moment refers to ankle plantarflexion, knee extension, hip flexion, and hip abduction. (TIFF) [file pcbi.1011837.s001.tiff]

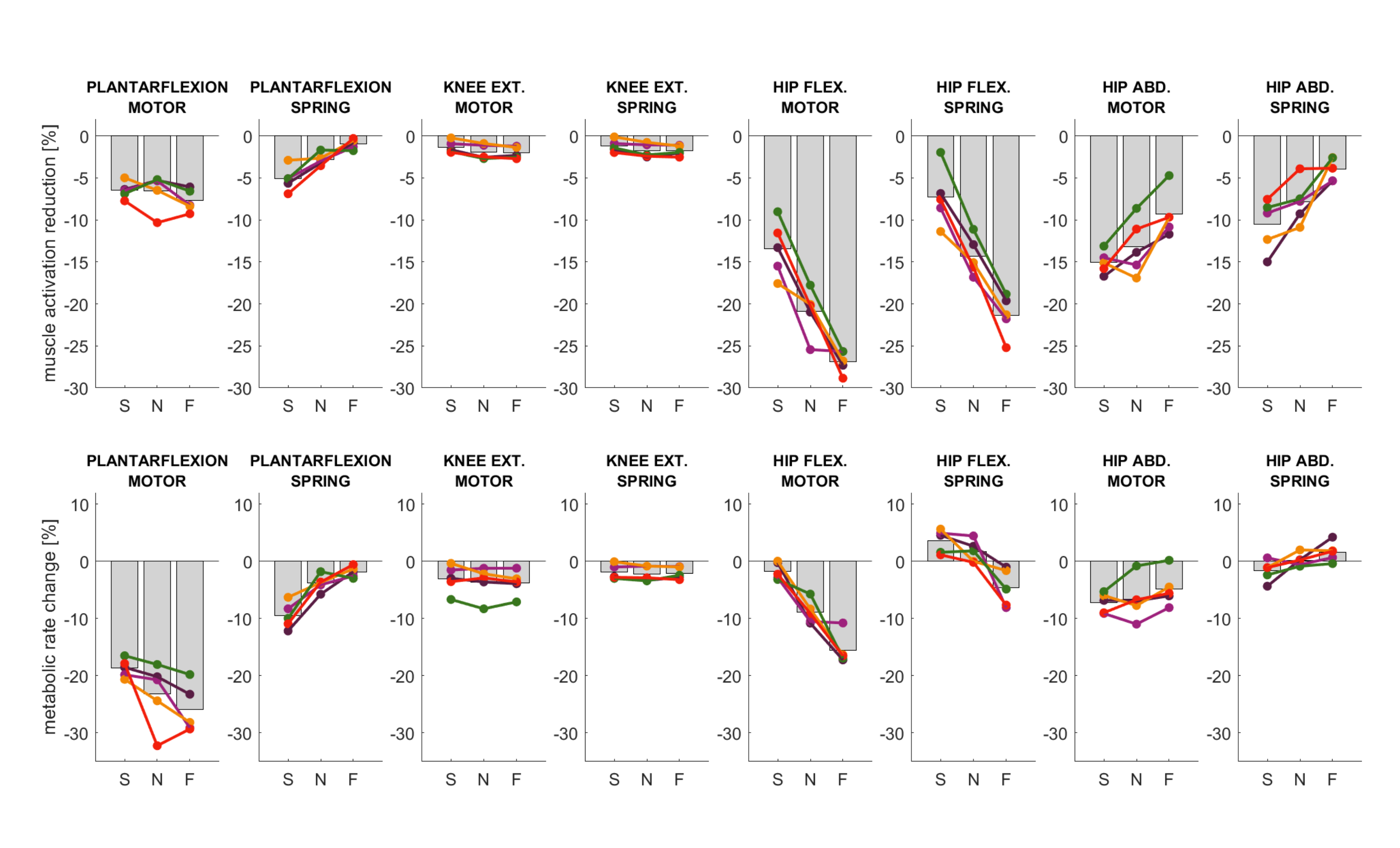

Supplement: S2 Fig — Change in metabolic rates vs. reduction of muscle activations per each subject, shown as % of unassisted conditions, at slow (S), normal (N), and fast (F) walking speeds with motor-based and spring-based assistance. Each color represents a different subject (average value of three gait cycles per subject). (TIFF) [file pcbi.1011837.s002.tiff]

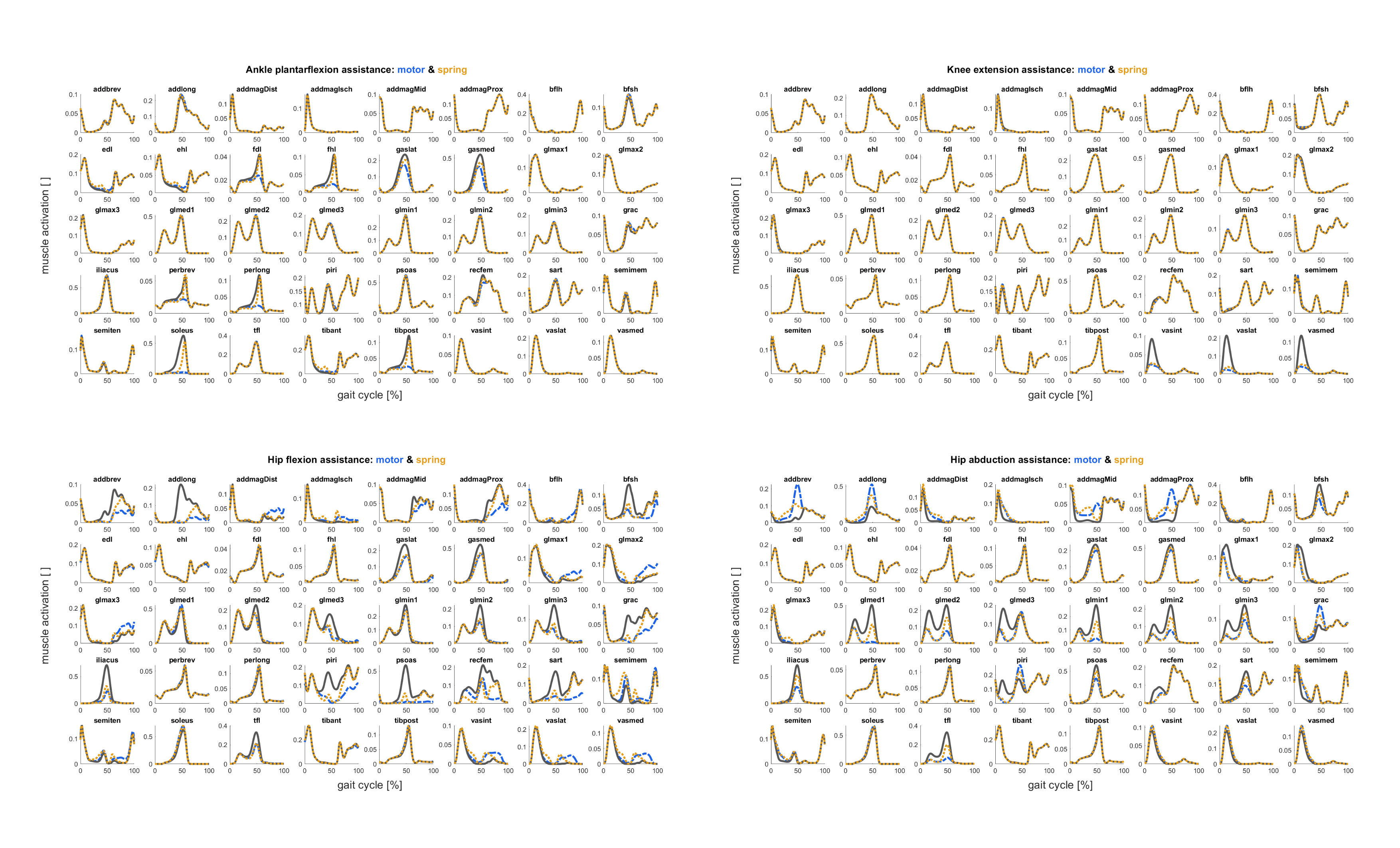

Supplement: S3 Fig — Average muscle activations in unassisted conditions (gray), with motor-based (blue) and spring-based (yellow) assistance of plantarflexion [upper left corner], knee extension [upper right corner], hip flexion [lower left corner], and hip abduction [lower right corner] at normal walking speed. Muscle names (plot titles) refer to their abbreviations in the musculoskeletal model. The figure illustrates average muscle values among all the subjects and gait cycles. (TIFF) [file pcbi.1011837.s003.tiff]

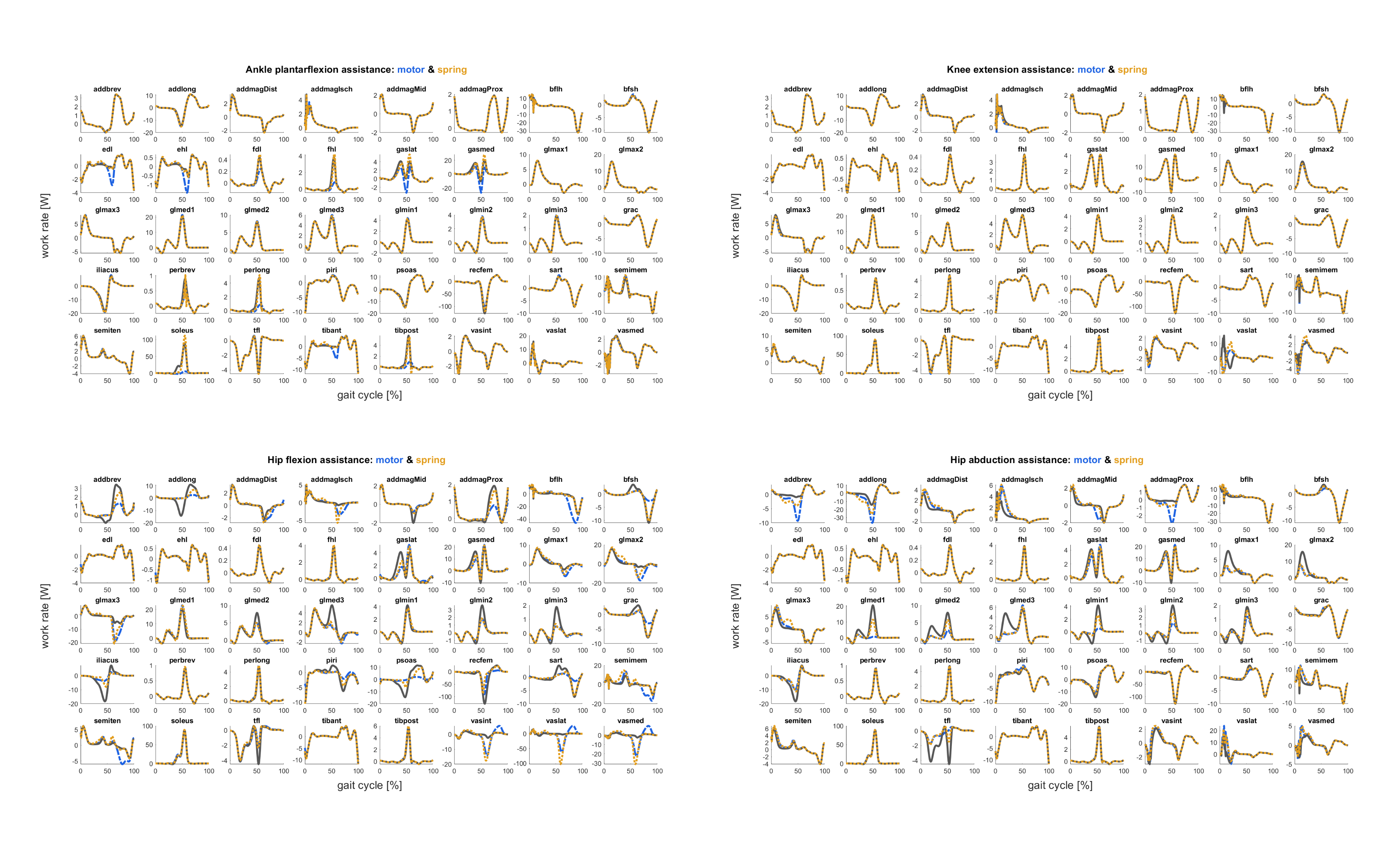

Supplement: S4 Fig — Average muscle work rate in normal conditions (gray) and with motor-based (blue) and spring-based (yellow) ankle plantarflexion [upper left corner], knee extension [upper right corner], hip flexion [lower left corner], and hip abduction [lower right corner] assistance at normal walking speed. Muscle names (plot titles) refer to their abbreviations in the musculoskeletal model. The figure illustrates average muscle values among all the subjects and gait cycles. (TIFF) [file pcbi.1011837.s004.tiff]

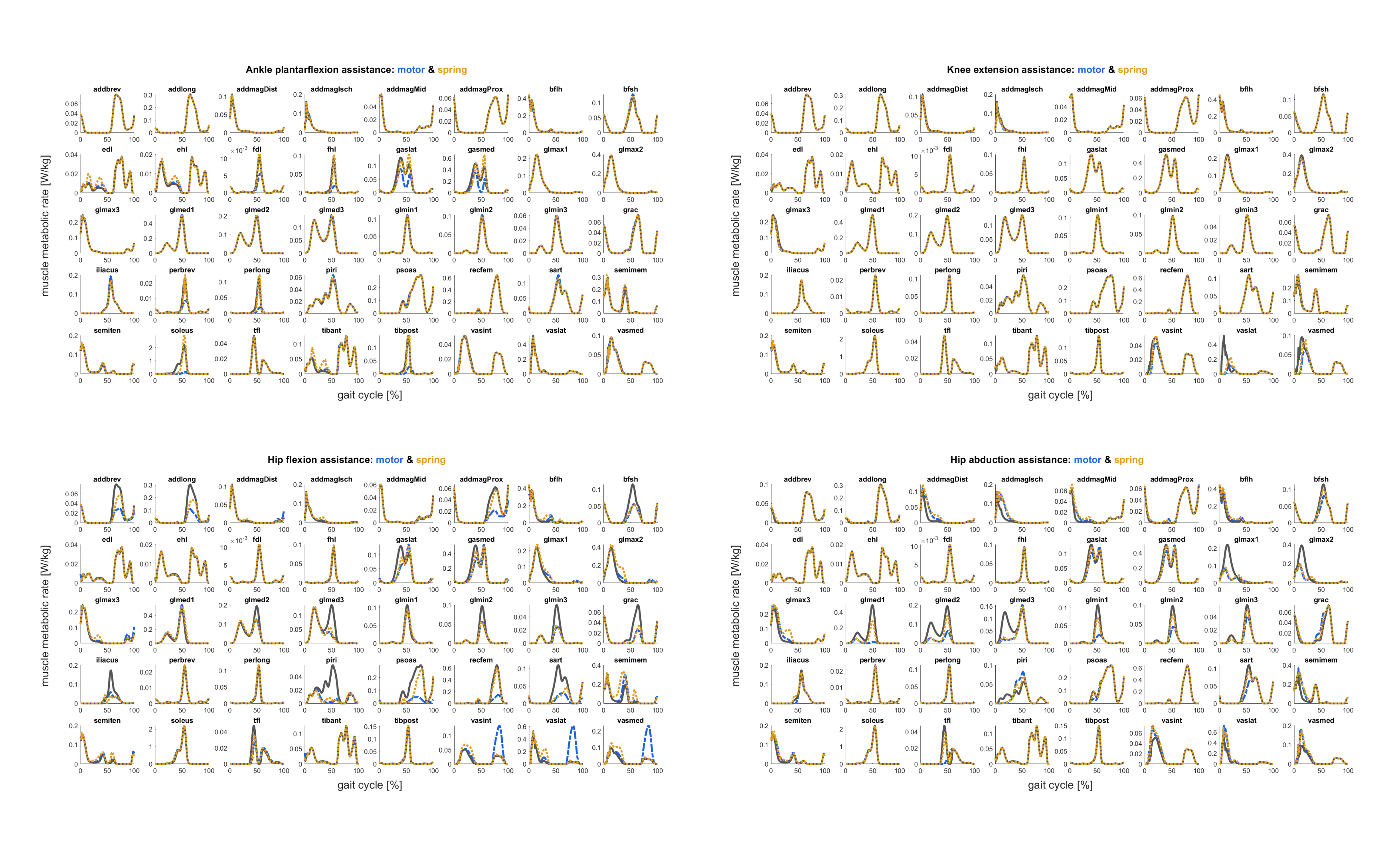

Supplement: S5 Fig — Average muscle metabolic rates (normalized to body mass) in normal conditions (gray) and with motor-based (blue) and spring-based (yellow) ankle plantarflexion [upper left corner], knee extension [upper right corner], hip flexion [lower left corner], and hip abduction [lower right corner] assistance at normal walking speed. Muscle names (plot titles) refer to their abbreviations in the musculoskeletal model. The figure illustrates average values among all the subjects and gait cycles. (TIFF) [file pcbi.1011837.s005.tiff]
